# How are genetic test results being used by Australian life insurers?

**DOI:** 10.1038/s41431-018-0198-z

**Published:** 2018-06-11

**Authors:** K. Barlow-Stewart, M. Liepins, A. Doble, M. Otlowski

**Affiliations:** 1 Sydney Medical School - Northern, University of Sydney Level 7 Kolling Institute, Royal North Shore Hospital, St Leonards, NSW 2065 Australia; 2 Consultant Actuary, 406/116 Belmont Rd, Mosman, NSW 2088 Australia; 3 Faculty of Law, College of Arts, Law and Education University of Tasmania, Private Bag 89, Hobart, TAS 7001 Australia

## Abstract

In Australia, the USA and many Asian countries the life insurance industry is self-regulated. Individuals must disclose genetic test results known to them in applications for new or updated policies including cover for critical care, income protection and death. There is limited information regarding how underwriting decisions are made for policies with such disclosures. The Australian Financial Services Council (FSC) provided de-identified data collected on applications with genetic test result disclosure from its life insurance member companies 2010–2013 to enable repetition of an independent examination undertaken of applications 1999-2003: age; gender; genetic condition; testing result; decision-maker; and insurance cover. Data was classified as to test result alone or additional other factors relevant to risk and decision. Where necessary, the FSC facilitated clarification by insurers. 345/548 applications related to adult-onset conditions. The genetic test result solely influenced the decision in 165/345 applications: positive (*n* = 23), negative (*n* = 139) and pending (*n* = 3). Detailed analyses of the decisions in each of these result categories are presented with specific details of 11 test cases. Policies with standard decisions were provided for all negative test results with evidence of reassessment of previous non-standard decisions and 20/23 positive results with recognition of risk reduction strategies. Disclosure of positive results for breast/ovarian cancer, Lynch syndrome and hereditary spastic paraplegia, and three pending results, generated non-standard decisions. The examination demonstrates some progress in addressing concerns in regard to utilisation of genetic test information but the self-regulatory system in Australia only goes some way in meeting internationally recommended best practice.

## Introduction

Underwriting describes the process whereby a life insurance company assesses the risk of claiming carried by an applicant in order to determine the cost charged for cover of that risk [1]. Life insurance products include cover for death; trauma that provides payment if a person is diagnosed with a specified serious illness or injury, including the major illnesses or injuries that will make a significant impact on a person’s life, such as cancer or a stroke; total and permanent disability (TPD) that pays a lump sum if the person becomes totally and permanently disabled; disability income (DI) that replaces the income lost due to a person’s inability to work due to injury or sickness; and business expenses (BE) [2]. The assessment of risk is informed by a number of factors that include age; gender; lifestyle; personal and family medical information; and the type of cover applied for [3]. Living Benefit policies such as cover for trauma, TPD and DI are associated with different chances of making a claim than pure death policies and correspondingly higher premiums are charged. The commercial nature of life insurance means that different companies apply different charges for similar products according to commercial-in-confidence information underpinned by an underwriting manual.

Depending on an applicant’s age, gender and other common influencing factors such as smoking, each company will either accept and offer a policy as standard and charge a standard premium or make a non-standard decision which may include a loading on the premium (higher charges), an exclusion of claim for particular conditions or the term of coverage may be limited. In accordance with the governing legislation, where policies are mutually rated, applicants are required to disclose all information known to them relevant to their risk, when asked by the insurer [4]. It is also a requirement for the insurers to assess the risk appropriately, using statistical or actuarial data where that is available or else using other information on which it is appropriate to rely, and also to take into account any medical surveillance or preventive health strategies adopted by the insurance applicant [5]. In Australia many of these requirements are formalised as Standards by the Financial Services Council (FSC) that represents almost 100% of Australian companies involved in the provision of life insurance policies [6]. Once a life insurance policy has been established it is guaranteed renewable: if premiums are paid, there is no requirement to disclose any changes to circumstances [4].

While an insurance applicant’s genetic information has long been inferred from their family history, the increased availability and decreasing costs of genetic testing means that genetic test results could increasingly contribute to the underwriting information that insurers seek. Internationally, the potential for genetic discrimination (GD), defined as adverse treatment on the basis of an individual’s actual or presumed genetic makeup, has been reported as a primary concern for individuals considering predictive genetic testing for adult onset conditions, specifically in relation to employment and insurance [7, 8]. Joly et al (2017a and 2017b) documented the range of responses to this concern that many countries have implemented to limit the potential for GD: moratoria, ethical guidance, self-regulation, sectoral prohibition, genetic exceptionalism, human rights and hybrids; while others have instead maintained the status quo [9, 10]. International insurance think-tanks have similarly been active in classifying the various approaches different countries have taken towards the use of genetic test information when underwriting life insurance policies [11]. The Australian life insurance industry is self-regulated as occurs in the USA and in many Asian countries [12, 13].

The Australian Government broadly accepted the recommendations in the report from an Inquiry held into the Protection of Human Genetic Information conducted by the Australian Law Reform Commission (ALRC) and the Australian Health Ethics Committee, 2000–2003, that, in relation to life insurance, the status quo should apply whereby applicants must disclose all information known to them that is relevant to their risk assessment [12]. Nevertheless, a number of recommendations were also made as a *quid pro quo* for this decision, the implementation of which was a matter for the FSC - formally known as Investment and Financial Services Association (IFSA) - and the Insurance Council of Australia [13]. Some of these recommendations have been incorporated into FSC self-regulation Standards that mandate the industry’s practice in regard to genetic testing and family history respectively: Standards 11 and 16 [14, 15]. These include that insurers should not request new genetic tests as part of a risk assessment but that any genetic test result known to the applicant needs to be disclosed and the insurer should provide reasons for adverse underwriting decisions. Insurers should also take into account surveillance and preventive strategies undertaken to reduce risk, ensure that risk assessment is underpinned where possible by relevant actuarial or statistical data and limit family history disclosure to first degree relatives only.

Internationally, there is debate about the widespread existence of GD in the life insurance sector, with significant variations between studies and dependent on the genetic condition, with a number of studies focussing on a single genetic condition [16]. It needs to be recognised however that evidence of GD is difficult to obtain with barriers expressed by consumers in regard to making complaints or accessing legal remedies [17]. While a number of studies have sought to investigate GD by obtaining data directly from insurers, policies that limit insurers collecting genetic test information make such investigations an increasing challenge [2, 18, 19].

However in Australia the FSC has been collecting information annually since 1998 from its members on all applications for a life insurance product where a genetic test result has been disclosed as per Standard 11 [20]. Analysis of this data collection provides a unique opportunity to understand underwriting practices in regard to genetic test result information. The Genetic Discrimination Project (GDP), a nationwide study conducted 2000-2005 that used a triangulated approach to investigate GD from both legal and social perspectives and with a verification component of consumer experiences, accessed the FSC data collected over the period 1999-2003 for independent examination for possible GD [2, 20]. Data was available for analysis in regard to 234/288 individuals who made applications for one or more insurance products where a genetic test result was disclosed. Genetic test results reported included hereditary haemochromatosis (HH, 71%), Huntington disease (HD, 12%) and breast/ovarian cancer (Bra/Ov, 6%); 67%% and 33% negative and positive results respectively were disclosed. Analyses indicated that the majority of the policy decisions that used genetic test results were informed by risks likely to be relevant to underwriting within a commercial environment, but identified three concerning cases involving breast and ovarian cancer where very broad exclusions were applied or there was a lack of recognition of prophylactic surgery to reduce risk.

In 2015, the FSC accepted an application to repeat the independent examination of newer data and compare the findings to (1) identify the volume of genetic test results disclosed in life insurance applications and (2) examine underwriting decisions in those applications where a positive or negative predictive genetic test result was the primary influencing factor.

## Materials and methods

### Data collection

The FSC provided researchers with de-identified data in regard to the applicant and the life insurance company involving all applications containing a genetic test result disclosure between April-2010 and September-2013 received by the FSC from its member insurance companies. The period was determined by the collection method to enable comparison to the data collected 1999–2003 as from October 2013 the survey form used to input the data by insurers was changed.

The data were provided in an Excel spreadsheet format, inclusive of: date of birth, gender and smoking status; the condition(s) for which the genetic test was undertaken and genetic test result(s); the cover applied for and the underwriting decision – whether standard or non-standard (i.e. loaded, limited, deferred or declined); the influencing factor(s) on a non-standard decision (genetic test result, other medical or non-medical reason); notes relevant to the decision; the status of the person(s) making the decision (senior underwriter, chief underwriter or medical officer); and sources of expertise consulted. Applications were for death, trauma, TPD, DI and/or BE cover.

### Clarification of data

Case-by-case review of insurer comments revealed inconsistencies between insurers in the classification of the genetic test result and other factors influencing the underwriting decision. It was therefore necessary to reclassify cases into the following categories: positive; negative; uninformative (no causative variant affecting function identified but testing done due to a family history); heterozygous carrier for an autosomal recessive condition; pending (genetic test initiated or intended and the result not available at the time of underwriting); and unknown (genetic test result not provided or unclear from the information provided). Cases were also classified as to whether applicants were asymptomatic or symptomatic for the condition or where other medical/non-medical factors were disclosed and influential to the underwriting decision.

### Data analysis

Through the lens of potential GD, only those cases where the genetic test result disclosed was by an individual who was asymptomatic for an adult onset condition at the time the application was made and where the result was the only influencing factor were included for examination of the underwriting decisions.

Where the actuarial and genetic counselling expertise on the research team required further assistance in regard to assessment of the risk associated with the genetic test result, underwriters and clinical geneticists or genetics experts were consulted as well as examination of the relevant literature. Clarification of cases with unclear results or unclear decisions was with the relevant insurance companies via the FSC.

Ethics approval for the study was granted by the University of Sydney’s HREC.

## Results

### The applicants

In all, 548 individuals disclosed a genetic test result in applications for a range of policies. Applicants were resident in all Australian States and Territories, 54% were male and the majority were aged 30–49 years.

### The insurers and decisions

Seven life insurance companies provided the data to the FSC. While the FSC has about 100 members, not all are life insurers that offer policies for cover relevant to GD where individuals are underwritten [6]. Many offer policies under an umbrella of “group insurance” within the Australian retirement fund system of superannuation where limited health information is required [3]. Two companies provided data in regard to over 50% of the applications. Given the mandatory requirement under *10.16* of Standard 11 Genetic Testing Policy, it would be expected that all companies provided data to the FSC where genetic test results were disclosed in applications [14].

**Fig. 1:**
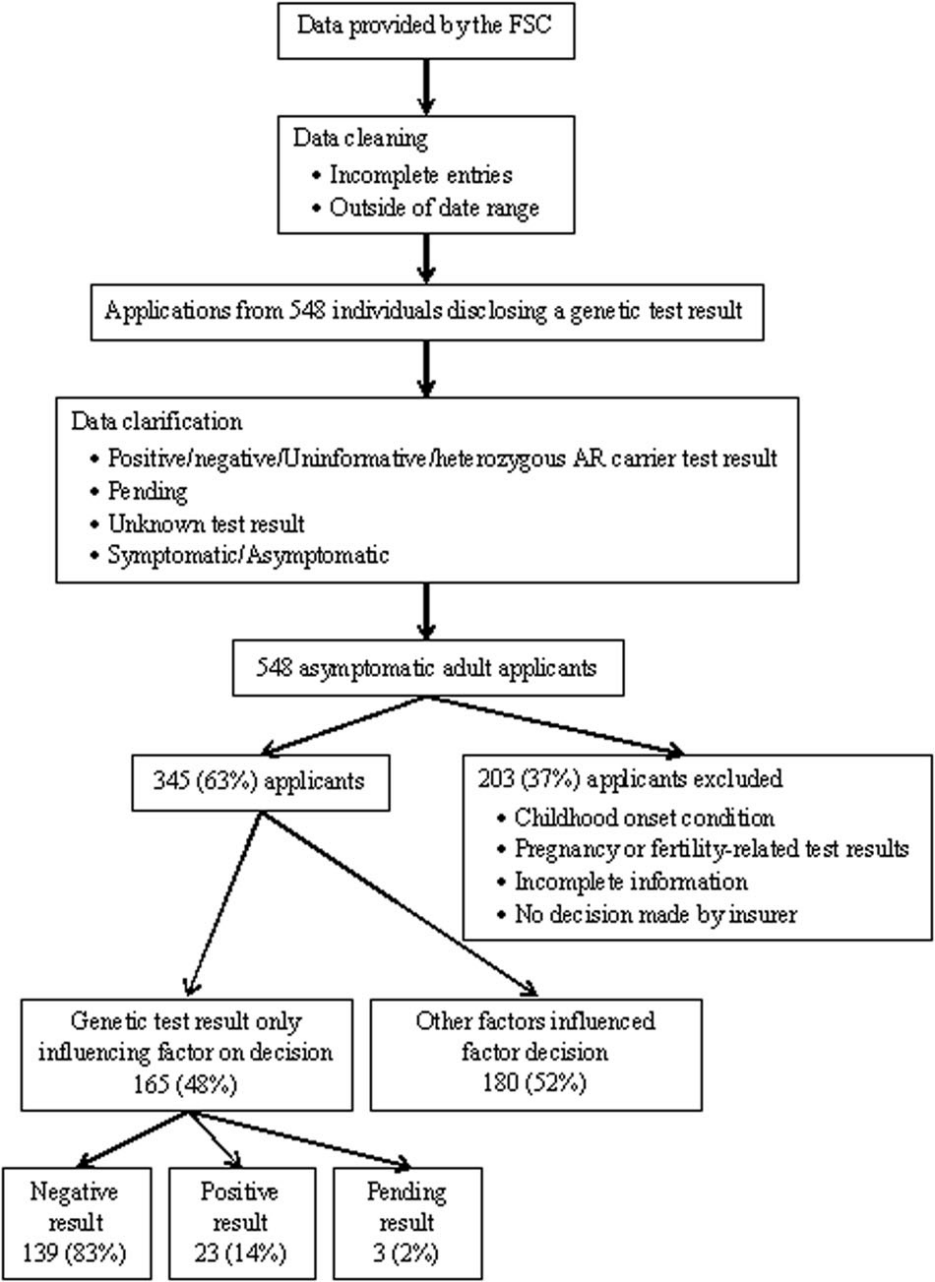
Data collection and analyses process

Of the 548 applications, 41% applied for cover only for death, trauma, TPD, DI or BE; 27% applied for two of these products; 23% for three; 8% for four; and 1% for all five products. Insurers made 1050 decisions in regard to these applications; four applications were withdrawn before a decision was made and for a further 57 applications a decision was still pending at the time of data collection (Table 1). Overall, 65% of the underwriting decisions were standard; 23% non-standard decisions with either premiums loaded, certain conditions excluded or the sum insured reduced; 2% deferred; and 10% declined. 203/548 (37%) were excluded: genetic test results relating to childhood onset conditions (*n* = 108); pregnancy/fertility (*n* = 43); incomplete information or no decision (*n* = 52) (Fig. 1). 345/548 (63%) applications that involved disclosure of a genetic test result for an adult onset condition were considered valid for analysis in terms of potential GD.

**Table 1:** Type of life insurance product requested by 548 applicants and resulting underwriting decisions

	Death	Trauma	TPD	DI	BE	Other	Total Decisions *n* (%)
	*n* (%)	*n* (%)	*n* (%)	*n* (%)	*n* (%)	*n* (%)	*n* (%)
Standard decision	287 (78)	156 (71)	118 (52)	103 (49)	4 (67)	10 (59)	678 (65)
Loading/exclusion/reduced sum insured	52 (4)	40 (18)	74 (33)	70 (34)	2 (33)	4 (24)	242 (23)
Deferred	8 (2)	2 (1)	7 (3)	9 (4)	0	0	26 (2)
Declined	23 (6)	22(10)	28 (12)	28 (14)	0	3 (17)	104 (10)
	370 (34)	220 (21)	227 (22)	210 (20)	6 (1)	17 (2)	1050
Withdrawn	1	1	1	1	0	0	4 (7)
Pending	19	9	12	17	0	0	57 (93)
Total							1111

*TPD* total and permanent disability, *DI* disability income cover, *BE* business expenses.

### Genetic testing and adult-onset conditions

Genetic testing was most frequently reported for HH (58%); inherited susceptibility to cancer (15%); thrombophilia - Factor V Leiden and/ or prothrombin gene variants affecting function (9%); cardiovascular conditions (5%); neurodegenerative conditions (4%); and neuromuscular conditions (3%). Others included coeliac disease (*n* = 6); polycystic kidney disease (*n* = 4); ankylosing spondylitis (*n* = 2); diabetes insipidus (*n* = 2); Ehlers Danlos (*n* = 1); Gaucher Disease (*n* = 1); glaucoma (*n* = 1); lupus (*n* = 1); malignant hypothermia (*n* = 3); scleroderma (*n* = 1); and sensory neuropathy (*n* = 2) (Table 2).

**Table 2:** Genetic test result disclosures for adult onset conditions (*n* = 345)

Condition	Cases *n* (%)	Genetic test result only	Genetic test and/ or other factors relevant to decision
Hereditary haemochromatosis	200 (58)	92	108
Cancer	51 (15)	29	20
Thrombophilia	31 (9)	15	16
Cardiovascular	17 (5)	9	8
Neurodegenerative	13 (4)	10	3
Neuromuscular	9 (3)	6	3
Other conditions	24 (7)	4	20
Total	345	165 (47.8%)	180 (52.2%)

For 165/345 (47.8%), the genetic test result was the only influencing factor on the outcome of applications: individuals were asymptomatic at the time of genetic testing and no other medical or non-medical factors were cited (Table 2). The genetic test results were classified as negative in 139 (83%) cases; positive in 23 (14%) cases and pending in 3 (2%) cases (Table 3 and Fig. 1).

**Table 3:** Conditions where genetic tests disclosed with no additional medical or non-medical factors described as influential on the decision (*n* = 165)

Condition	Genetic test result		
	Negative	Positive	Pending
Hereditary haemochromatosis	78	14	
**Cancer**			
Breast/ ovarian	11	2	1
Colorectal	10	1	1
Cancer – other	3		
Thrombophilia	10	5	
**Cardiovascular**			
Cardiomyopathies	6		
CADASIL	1		
Aneurysm	2		
**Neurodegenerative**			
Huntington disease	7		1
Motor neurone disease	1		
Creutzfeldt-Jakob disease	1		
**Neuromuscular**			
Myotonic dystrophy	4		
Hereditary spastic paraplegia type 4		1	
Spinocerebellar ataxia type 1	1		
**Other conditions**	4		
Total	139 (84.2%)	23 (14.0%)	3 (1.8%)

For 11/165 cases, the reasons for the decisions made by the insurers were not self-evident from the data. Further investigations of these individual decisions were therefore carried out as test cases: clarification was facilitated by the FSC with the insurer (*n* = 6); and with experts in clinical genetics or underwriting (*n* = 5).

### Decisions where a genetic test result was the only factor influencing outcome

#### Negative for the family variant affecting function (*n* = 139)

In all cases the underwriting decision was standard for all types of insurance product applied for. Conditions included HH, cancer predisposition, thrombophilia, cardiovascular, neurodegenerative, neuromuscular and others (Table 3 and Fig. 1).

*Test cases 1, 2, 3 and 4*. In four cases the result engendered a reversal to a standard decision from an adverse decision previously made on the basis of family history: Bra/Ov cancer (*n* = 2), colorectal cancer (*n* = 1), and *MYH*7 gene associated with hypertrophic cardiomyopathy (*n* = 1). Details were only provided for the latter: reversal of standard death, trauma, TPD and DI.

#### Positive for a variant affecting function (*n* = 23)

Twenty individuals (11 males/9 females) received standard decisions for all products they applied for: HH (14/14), thrombophilia (5/5) and Bra/Ov cancer (1/4) (Table 4). For HH and the thrombophilias, treatment instituted affected the decision: venesection to reduce iron levels and anticoagulants to prevent deep vein thrombosis respectively. *Test case 5*. The 33-year old female non-smoker applicant who disclosed a positive test result for a *BRCA1* gene variant affecting function received a standard decision. FSC clarification revealed that risk reduction surgery had removed her risk.

**Table 4:** Underwriting decisions for applications with positive genetic test results

Condition	Standard decision (male/female)	Non-standard decision (male/female)
Hereditary haemochromatosis	14 (10 males/4 females)	
Cancer		
- Breast and ovarian	1 (female)	1 (female)
- Lynch syndrome		1 (female)
Thrombophilia	5 (1 male/4 females)	
Neuromuscular		
- Hereditary spastic paraplegia type 4		1 (female)
Total	20	3

In three other cases the decision was non-standard (Table 4). *Test case 6*. The non-standard decision made by the Senior Underwriter and Chief Medical Officer for a 24-year old female non-smoker positive for the *BRCA2* gene variant who applied for DI was cover with exclusion of a claim for any cancer. *Test case 7*. The non-standard decision made by the Senior Underwriter and Chief Medical Officer in consultation with “Other medical doctor or geneticist and Reinsurance company” for a 45-year old female non-smoker positive for a gene associated with Lynch syndrome (that was described in the data as *MLH6)* who applied for trauma, TPD and DI was given cover with exclusions of a claim for any cancer on the trauma and TPD and loading on the DI cover. As the mismatch repair genes that are associated with Lynch syndrome include *MLH1* or *MSH6*, but not *MLH6*, a query was sent to the insurer via the FSC. Unfortunately, the insurer was unable to locate the actual file but noted that the data in regard to the gene name *(MLH6)* would have been imported from the information supplied by the applicant on the personal statement and also responded in a general way to the issue of the exclusion. It was stated that a cancer exclusion clause, or an exclusion of all kinds of malignant tumours, was felt to be necessary, as a narrower exclusion would not be satisfactory for the insurer, because of the difficulty of proving whether or not a subsequent tumour is a direct consequence of the particular gene variant affecting function tested for. It was also stated that metastases would raise too many medical and legal questions in attributing their cause and effect. This appears to have been a commercial judgement by the insurer of the possible financial risks if a subsequent claim occurred. *Test case 8*. The non-standard decision made by the Senior Underwriter and Chief Medical Officer for a 33 year old female positive for *SPAST* for SPG4 (hereditary spastic paraplegia type 4) was to decline death, trauma and TPD cover.

#### Pending results or test planned (*n* = 3)

*Test cases 9 and 10*. A 43-year old female non-smoker who was waiting for test results for Bra/Ov cancer who applied for cover for death, trauma, TPD and DI and a 31-year old female non-smoker applicant for cover for DI waiting for colorectal cancer geteic test results were both declined cover altogether. *Test case 11.* A 33-year old male, who planned on taking a genetic test for HD applied for death and TPD cover and was declined. The underwriter commented that “client about to undergo genetic testing due to family history”.

## Discussion

In 2012, about 20% of Australians had policies in place for personal life insurance products, a level that is likely to be the same today [21, 22]. Concerns have been raised about this low figure given the importance of life insurance in future planning and support given that in Australia life insurance is a social good, as has been noted is the case in Europe [23, 24]. In 2005, of 455,000 Australian insurance applications processed, 400 involved people who had genetic tests [22]. The landscape of genetic testing is broadening to genomic testing and in the last few years has become cheaper, faster and more accessible, underscoring the need to understand how insurers will increasingly underwrite genetic and genomic test results that are disclosed in applications for life insurance [5].

In the last 10 years there has been a 90% increase in the total number of applications involving genetic test disclosures, perhaps reflecting this increased availability of genetic testing. Nevertheless those disclosing genetic test results are still a very small proportion of the total number of insurance applications made in Australia each year. Similar to 10 years ago, the majority of results related to genetic testing for HH, perhaps as it is one of the few DNA tests where the cost is covered by Australia’s national health insurance system called Medicare [2, 25]. The proportion of applications where the genetic test result is the only influencing factor on the underwriting decision were also about the same at around only half of all applications. Yet consumers may perceive that the genetic test result was the sole reason for the decision or at least a major influence - perhaps perceiving GD - so this underscores the importance of insurers’ giving applicants the reasons for non-standard decisions [12]. The data did not provide information as to whether such notification had been issued to applicants.

The proportion of negative test results disclosed had increased however. A negative test result for a family variant affecting function removes any risk inferred from a family history. The resulting expected reversal of non-standard decision to standard was demonstrated in the data in both this and the previous study. Importantly, information used by Genetics Services throughout Australia highlights informing patients of the importance of contacting insurers to have non-standard decisions made on the basis of family history reversed if a negative result is found [26]. Perhaps increased awareness and advocacy by genetic counsellors and clinicians in this regard has contributed to the observed change.

Further, the number of applications where a positive test result was disclosed almost halved. Where treatment or risk reducing strategies were possible and implemented these were recognised and standard decisions offered. However, in two cases (Bra/Ov cancer and Lynch syndrome), broad exclusions for any cancer claim were imposed, rather than exclusion of those cancers associated with the test result. This also occurred in the previous study [2] and such broad exclusions remain of concern to patients and their health professionals. While in the case of Lynch syndrome the insurer argued that the risk imposed for the variety of cancers associated was too high for targeted exclusion, similar arguments would not apply in regard to the *BRCA* test result. It is possible that the positive test result for the applicant at risk for hereditary spastic paraplegia type 4 engendered a level of risk that would be considered too high for a commercial contract resulting in denial of cover [27].

Denial of cover was also seen where results were pending or a test planned. An underwriting expert consulted as to why the decisions were not simply deferred until results were available, noted that standard practice in Australian underwriting is always to make a definite decision and provide certainty. Deferral for six months, for example, may therefore be perceived as a non-decision. This approach to underwriting is applied to all cases with pending medical activity – not just where genetic testing is about to happen or awaiting results – following an Australian landmark legal case relating to the remedies available to a life insurance company when considering non-disclosure [4, 28]. The initial decision to decline does not preclude the applicant from re-applying at a later date, when the pending genetic testing has been completed but it was not possible to assess how clearly the option of reapplying later was set out to the applicants.

### Implications for genetic counselling practice

All Australian genetic testing consent forms include a clause that the genetic test result may affect the ability of individuals undergoing genetic tests to obtain some types of insurance [29]. The Human Genetics Society of Australasia (HGSA) has produced guidelines for pre-test genetic counselling that recommend use of educational materials to support these explanations “such as that developed by NSW Health’s Centre for Genetics Education” [3, 26, 30]. Their fact sheet describes an individual’s disclosure obligations including known personal genetic test results; heath information about first degree relatives although not their genetic test result; possible outcomes of an insurance application; considerations by the underwriter in addition to the test result, for example, risk reduction strategies; negotiating with insurers; and dispute resolution [26].

A possible consequence of this increased awareness of those considering genetic testing is taking out their insurance before having the test, thereby avoiding any disclosure of a positive test result resulting in an adverse decision or enabling a reversal if the result is negative for a family variant affecting function as shown in the data. This may be a factor in the reduction of positive results disclosed reported in the data as the insurance is guaranteed renewable if they take it out before they have their test. Importantly, this strategy of genetic counsellors informing individuals of the insurance implications of genetic testing and potential to minimise difficulties by putting appropriate insurance in place before undertaking genetic testing is both ethically and legally justifiable. It does not amount to adverse selection by individuals against insurers because at the time that an individual applies for their insurance they do not have greater knowledge than the insurer (as family history would in any event need to be disclosed). In the event of positive genetic test results, the insured would have no reason to re-contact the insurer because as noted, once a life insurance policy is secured it is guaranteed renewable, and there is no obligation on the insured to advise of a change in circumstances.

### Limitations

The researchers could not independently check that all applications were captured for the period of Apr-2010 to Sep-2013, and hence could not rule out the possibility of under-reporting, although the mandatory requirements under FSC Standard 11 should act against underreporting. In fact, Barlow-Stewart et al. identified that there was some under-reporting of cases of adverse underwriting in the data collected 1999–2003 [20]. It must also be recognised that 50% of cases came from only two companies which may have introduced a bias and making the results not reflective of practice of the industry as a whole. This may also have impacted on the analyses regarding the volume of applications with disclosure of genetic tests results and limit the comparison between the data sets over the 10 years of reporting.

The data did not include whether the genetic test had been undertaken in the context of a research study or in a clinical setting. The genetic test result data available was also limited in lack of details of the variant affecting function involved that may have informed more precise risk assessment. Additionally, the number of positive genetic test results reported is quite small and so the researchers are very limited in their ability to examine the underwriting decisions in terms of possible GD. A large number of individuals applied simultaneously for a range of product types. The analysis did not determine whether cover may have been declined for one product but accepted for a different product type (even with modified terms of coverage).

### Further research

Understanding the views of insurers is essential in any investigation of GD. Of equal importance will be to survey individuals who have had genetic testing, or who have considered undertaking genetic testing, about their perceptions of the impact of this genetic testing on their life insurance eligibility. It would also be of great interest to conduct a similar verification study to that of Barlow-Stewart et al. in regard to applications received 2010-2013 to investigate any under-reporting [20].

## Conclusion

The underwriting decisions documented reflect an assessment of risk by Australian life insurers that is likely reflective of a commercial interpretation and impact. However, the landscape of testing is changing, in the context of the broad availability of genetic and genomic testing with decreasing costs, resulting in increased complexity of underwriting. The HGSA has been proactive in urging the insurance industry to not require disclosure of genetic testing undertaken as part of a research project as it may impact upon research participation and to implement a moratorium on the use of predictive genetic information pending improved actuarial estimates of the impact of such information on adverse selection [31]. Similar concerns were identified at a meeting of multidisciplinary experts in Quebec, Canada in 2012, and a number of recommendations were made for best practice going forward [24]. In regard to these, the following summarises where Australia is currently placed: (1) That life insurance policies be available covering a minimal (ceiling) amount at an affordable rate and with no health questions asked (including about genomics) is partially addressed at a limited level through the Australian retirement Superannuation system [32]. (2) The recommendation for an establishment of an Independent third party (ombudsman/body) with expertise in both genomics and personal insurance underwriting to address complaints has not been met [6]. However the data provided does not indicate whether or not insurers are providing reasons to inform complaints. (3) Australia meets the recommendation that information material and frequently asked questions about genomics and underwriting be developed [26]. (4) While through the FSC Standards openly accessible reference documents regarding the practices of their members on the use of genetic test information are in place, a recent update to Standard 11 means there is overall decline in the protection provided [14]. (5) The independent analysis documented here goes some way in addressing the recommendation that the industry have an independent third party perform regular audits of practice. However, the *ad hoc* basis on which this has been done, and the lack of published reports since 2005 of the FSC data, are caveats.
